# Colorectal-Vaginal Fistulas: Imaging and Novel Interventional Treatment Modalities

**DOI:** 10.3390/jcm7040087

**Published:** 2018-04-22

**Authors:** M-Grace Knuttinen, Johnny Yi, Paul Magtibay, Christina T. Miller, Sadeer Alzubaidi, Sailendra Naidu, Rahmi Oklu, J. Scott Kriegshauser, Winnie A. Mar

**Affiliations:** Mayo Clinic Arizona; Phoenix, AZ 85054 USA; Yi.Johnny@mayo.edu (J.Y.); Magtibay.Paul@mayo.edu (P.M.); cmille75@UIC.EDU (C.T.M.); Alzubaidi.Sadeer@mayo.edu (S.A.); Naidu.sailen@mayo.edu (S.N.); Oklu.rahmi@mayo.edu (R.O.); Kriegshauser.Scott@mayo.edu (J.S.K.); wmar@uic.edu(W.A.M.)

**Keywords:** colorectal-vaginal fistula, fistula, percutaneous fistula repair

## Abstract

Colovaginal and/or rectovaginal fistulas cause significant and distressing symptoms, including vaginitis, passage of flatus/feces through the vagina, and painful skin excoriation. These fistulas can be a challenging condition to treat. Although most fistulas can be treated with surgical repair, for those patients who are not operative candidates, limited options remain. As minimally-invasive interventional techniques have evolved, the possibility of fistula occlusion has enriched the therapeutic armamentarium for the treatment of these complex patients. In order to offer optimal treatment options to these patients, it is important to understand the imaging and anatomical features which may appropriately guide the surgeon and/or interventional radiologist during pre-procedural planning.

## 1. Review of Current Literature on Vaginal Fistulas

Vaginal fistulas account for some of the most distressing symptoms seen by clinicians today. The symptomatology of vaginal fistulas is related to the type of fistula; these include rectovaginal, anovaginal, colovaginal, enterovaginal, vesicovaginal, ureterovaginal, and urethrovaginal fistulas, with the two most common types reported as being vesicovaginal and rectovaginal [1]. The etiology of vaginal fistulas includes obstetrical complications, inflammatory bowel disease, post-surgical causes, pelvic malignancies, trauma, infection, congenital conditions, and radiation effects [2].

The prevalence of fistulas is poorly delineated in the literature, and varies widely in regards to etiology. Worldwide, the most common types of fistulas are vesicovaginal and rectovaginal due to obstetric causes [3]. In developing countries, the prevalence of vaginal fistulas may be as high as 1 in 1000 women, and occur as a result of prolonged or obstructed labor [4]. This is thought to be a result of tissue necrosis in the pelvis, caused by tissue entrapment between fetal parts and the bony pelvis [5].

In industrialized countries, vaginal fistulas are more commonly iatrogenic, resulting from surgical intervention or radiation therapy [4]. The most common iatrogenic cause is surgery following obstetric complications or gynecologic procedures, predominantly hysterectomies, with the most common type thought to be vesicovaginal fistulas [6]. Abdominal hysterectomies are associated with the highest risk of fistula formation, and the risk is increased in women over the age of 50 [1]. Other risk factors include smoking, diabetes, obesity, and a previous history of irradiation [1].

In the setting of pelvic malignancies, tumors may cause local tissue destruction resulting in fistula formation [7]. Radiation can lead to fistula formation due to obliterative endarteritis and chronic ischemia. After radiation treatment of cervical cancer, the incidence of vesicovaginal and rectovaginal fistulas varies from 1% to 8%, respectively [2]. Another common cause of fistulas in the developed world is Crohn’s disease; up to 10% of those with Crohn’s disease will develop some type of fistula, with anovaginal and rectovaginal fistulas accounting for approximately 9% of these cases [8].

The accurate diagnosis of vaginal fistulas begins with a detailed history and careful physical examination of the patient. Affected patients experience a myriad assortment of major complaints, which may include the passage of feces or flatus from the vagina, recurrent urinary tract infections, vaginitis, vaginal bleeding, and/or malodorous vaginal discharge [9,10]. Low-lying rectovaginal and anovaginal fistulas may be directly observed on anoscopic and speculum examination, however the majority of fistulas will not be clinically localized on physical examination. Historically, vaginography and barium enema examinations have been used to detect vaginal fistulas communicating with the gastrointestinal tract, while cystoscopy and urethrography have been used to detect communications within the genitourinary system [2,11]. Water-soluble contrast is preferred, as the risks of using barium as a contrast agent include venous intravasation and peritoneal spillage, a potential pitfall of fluoroscopic diagnosis. However, because of the dilution of water-soluble contrast agents, a negative study performed with water-soluble contrast can be followed by a barium study when clinical suspicion remains high [12]. Endoluminal sonography has also been used with variable success, and has been shown to be superior to clinical examination alone [10,13]. Currently, the preferable modalities of choice for pelvic fistula evaluation are magnetic resonance (MR) and multidetector computed tomography (CT) for patients unable to get an MRI, with the goal of imaging being to provide the clinician with a specific anatomic site and fistula course.

MR imaging may be beneficial in conjunction with conventional surgical evaluation in certain specific situations, such as discriminating between simple and complex perianal fistulas [13,14]. Complex anal fistulas include tracks extending above the dentate line, multiple fistulous tracts, anovaginal fistulas, and fistulas associated with Crohn’s disease. MR imaging can offer delineation of the anal sphincter complex as well as the surrounding pelvic organs. In one study of patients with recurrent anal fistulas, MR imaging findings acted on by surgeons led to a 75% reduction in the recurrence of anal fistulas [10]. MR imaging is also advantageous given its multiplanar imaging capabilities. On T2-weighted imaging, fistulas usually appear as high signal intensity, fluid-filled communications to the vagina [1]. Specifically, fast spin-echo heavily T2-weighted images are the key sequences. On both T2-weighted and inversion recovery images, the posterior wall of the vagina and anterior wall of the rectum normally appear as a low signal intensity margin allowing clear visualization of T2 hyperintense fistulous tracts [15]. If a tract is air-filled, it will produce low signal intensity on MR images. Rectovaginal fistulas may demonstrate interruptions of the vaginal muscularis and rectum, with discontinuity of the intervening fat plane [16] In one study, the use of intravenous gadolinium-based contrast was deemed necessary in differentiating a pus-filled active fistula from a healing tract undergoing fibrosis; this is because active fistulas only demonstrate thin wall enhancement, while granulation tissue in a healing tract also has central enhancement [17].

Contrast-enhanced CT imaging is a useful alternative to MR imaging, specifically in patients who cannot tolerate MR imaging [2]. Although major studies are lacking in the literature, the sensitivity of contrast-enhanced CT in the diagnosis of vaginal fistulas may be greater than 60% [18]. Both intravenous and oral contrast should be administered if possible, given the increased accuracy of delayed contrast-enhanced CT images [19,20]. Imaging 40–60 min after ingestion has been shown to be both diagnostically reasonable and time efficient [18]. Three-dimensional reconstructions are helpful, not only in delineating anatomy, but also in surgical/interventional planning. On a CT urogram, ureterovaginal fistulas may appear as a faint direct tract outlined by contrast material, as well as a resultant opacification of the vagina [19]. Oral contrast is clearly advantageous in the evaluation of rectovaginal or anovaginal fistulas, not only in delineating a tract, but also in the identification of focal areas of bowel-wall thickening. Current protocol recommendations include an initial unenhanced CT followed by contrast-enhanced imaging during the portal and excretory phases with a split bolus [2].

Once diagnosis has been achieved, the management of patients with vaginal fistulas is as much determined by their etiology as by physical factors, including the size, location, and complexity of the tract. Given the overlap of this problem with fields such as obstetrics/gynecology, urology, and colorectal surgery, a multidisciplinary approach may be necessary for effective treatment. Although conservative management has been utilized in some cases, the vast majority of patients are treated surgically, with a new and evolving subset of patients receiving novel treatment with interventional assistance.

## 2. Surgical Management of Colovaginal and Rectovaginal Fistulas

Surgical correction of colovaginal and rectovaginal fistulas requires an understanding of the size, location, and etiology of the fistula. These factors allow the surgeon to counsel the patient as to the best approach to treatment, and the expected success following surgery. In reproductive-age women, the most common etiology for rectovaginal fistulas is obstetrical trauma, which physically would be identified low in the vaginal canal. In older women, fistula formation is usually due to an inflammatory process, such as diverticulitis or Crohn’s disease. Identifying the exact location of the fistula, which can be identified on imaging, is critical in order to decide the approach to surgery, whether it be transvaginal or transabdominal.

### 2.1. Low Rectovaginal Fistulas

Low rectovaginal fistulas are most likely to be caused by obstetrical trauma, but can also be due to inflammatory processes, such as Crohn’s disease. Preoperatively, an endoanal ultrasound can assess the integrity of the anal sphincter, as concomitant sphincteroplasty can be performed if necessary. Furthermore, it is important to assess a patient’s symptoms, as fecal incontinence has been reported in up to 48% of patients with rectovaginal fistulas caused by obstetrical trauma [21]. This high rate of fecal incontinence may correlate to unrecognized third or fourth degree lacerationw involving the anal sphincter. On physical examination, careful visualization of the posterior vaginal wall should be performed. Obstetrical injuries would likely be seen in the distal third of the vagina. If symptoms are consistent, but no fistulous tract is seen, dilute methylene blue can be instilled into the rectum, and a tampon placed into the vagina. Blue staining of the tampon can confirm the fistulous tract.

**Figure 1:**
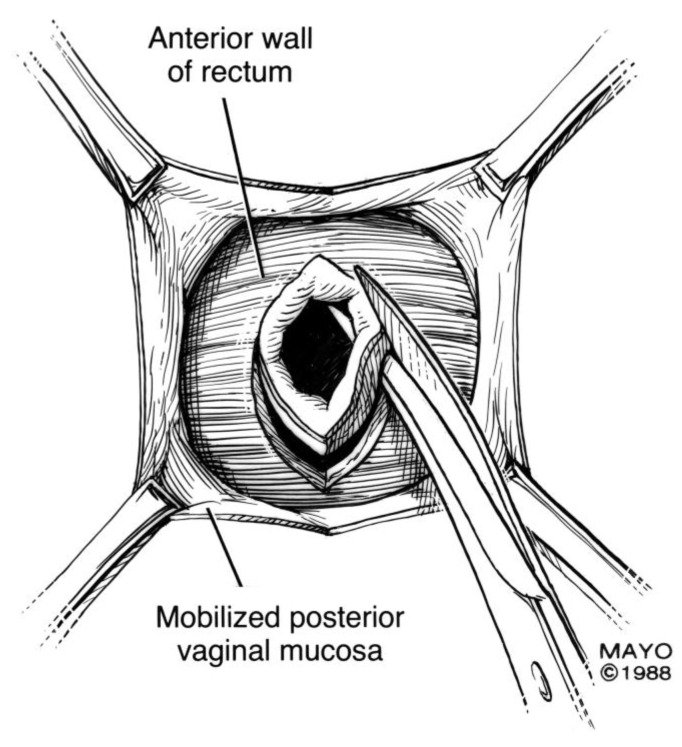
Anatomical illustration demonstrating technique of surgical repair of low rectovaginal fistulas.

**Figure 2:**
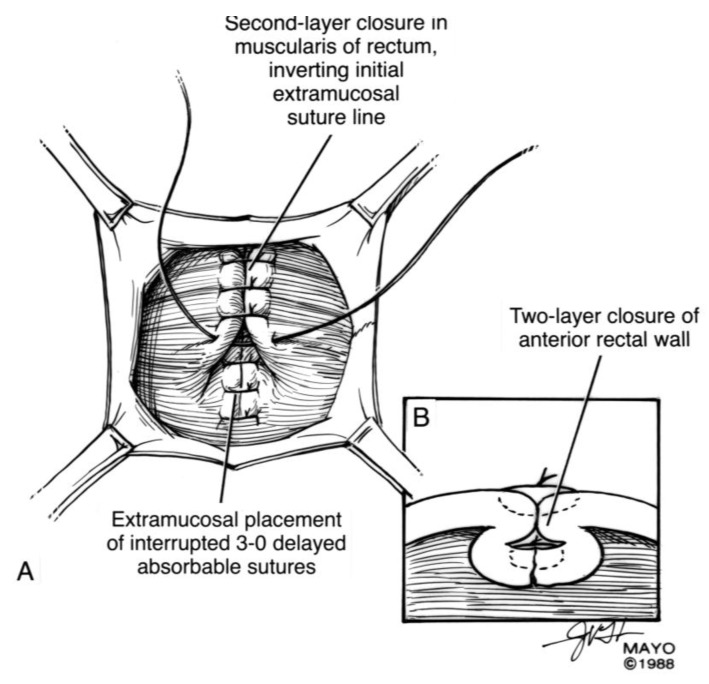
Anatomical illustration demonstrating technique of surgical repair of low rectovaginal fistulas.

**Figure 3:**
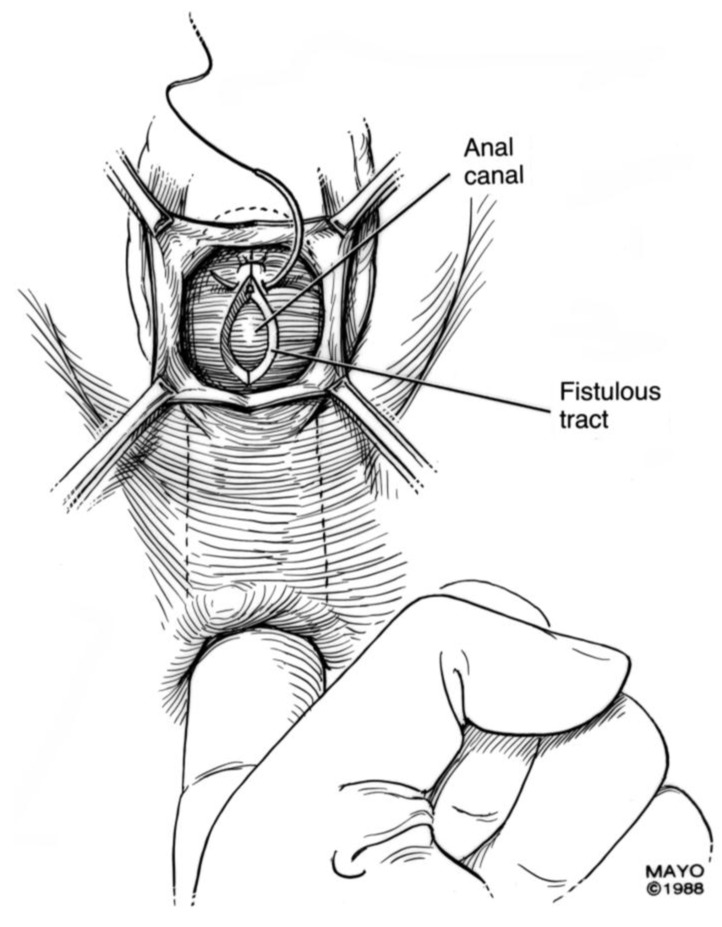
Anatomical illustration demonstrating technique of surgical repair of low rectovaginal fistulas.

**Figure 4:**
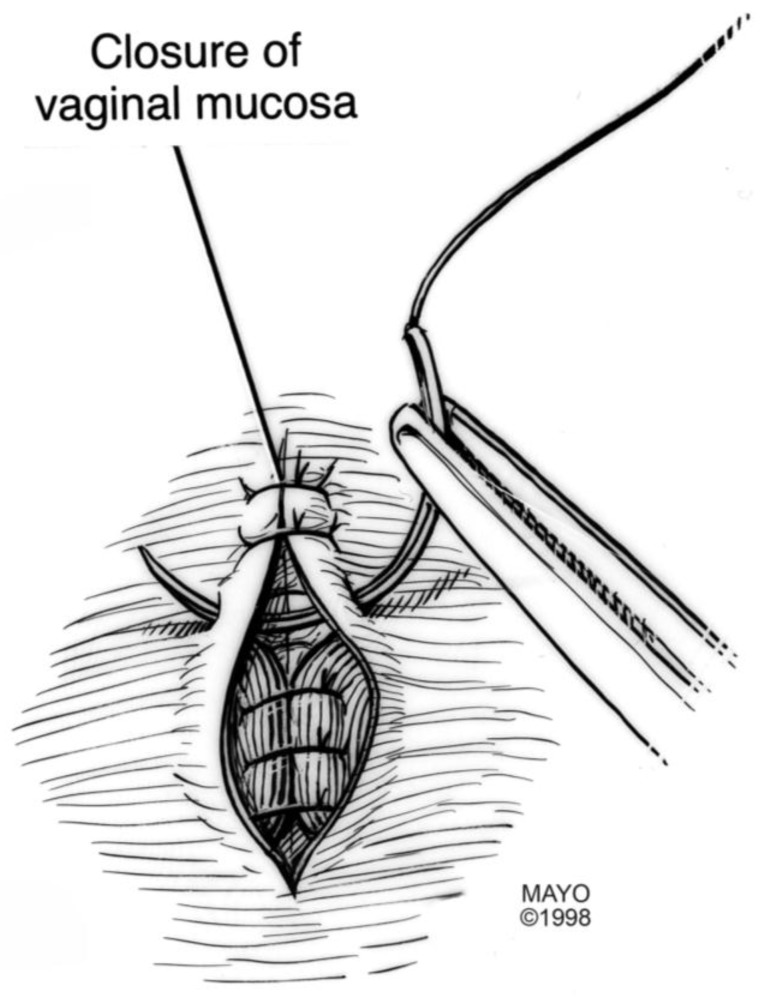
Anatomical illustration demonstrating technique of surgical repair of low rectovaginal fistulas.

For surgical correction of low rectovaginal fistulas, we prefer a vaginal approach, excising the fistulous tract with a multilayer closure. This can be performed in conjunction with an anal sphincteroplasty, if an obstetric anal sphincter injury is detected on an endoanal ultrasound. An enema can be performed either the night before or on the morning of surgery to empty the lower colon. Appropriate broad-spectrum antibiotics should be given perioperatively. Under general anesthesia, a thin metal probe can be inserted through the fistulous tract for identification. If the fistula involves the anal sphincter, or if sphincteroplasty is planned, an episioproctotomy can be intentionally performed along the midline of the posterior vaginal wall to the level of the fistula. If the external anal sphincter is intact, an incision can be made superficially in the midline of the posterior vaginal epithelium, avoiding separation of the sphincter. The fistula is excised until bleeding margins are visualized, indicating healthy rectal mucosa. The rectal submucosa and muscularis is reapproximated using a delayed absorbable suture (Figure 1, Figure 2, Figure 3 and Figure 4). Multiple layers of the overlying connective tissue are imbricated over the initial suture line.

The external anal sphincter is then reapproximated using either an end-to-end or overlapping technique with a delayed absorbable suture [22,23,24]. Care is also taken to reapproximate the internal anal sphincter, to the depth of the external anal sphincter. Rebuilding the perineal body with further layers over the rectal suture line provides strength to the repair, prior to reapproximating the vaginal epithelium. Finally, the vaginal incision is closed using a delayed absorbable suture in the midline, incorporating the perineal skin closure.

Postoperatively, patients are managed with soft, but not liquid stool, using laxatives and stool softeners as necessary. Enemas should not be used during the healing phase. Prolonged antibiotics are not necessary, but pelvic rest avoiding vaginal or anal intercourse is important to allow healing of the area for six to eight weeks. Sitz baths are helpful to clean the perineum without prolonged soaking, and incisions should be monitored for signs or symptoms of infection.

### 2.2. High Rectovaginal and Colovaginal Fistulas

An abdominal approach to rectovaginal and colovaginal fistula repair is considered if there is underlying colon pathology that needs to be addressed, if a diverting stoma is needed, or if the fistula is too high to reach vaginally for adequate exposure. The most likely scenario of a high colovaginal fistula is in an older patient with prior diverticular disease. These fistulas can often be visualized on pelvic examination in the left apex of the vagina, and symptoms include the passing of flatus or fecal material through the vagina. If a prior hysterectomy has been performed, involvement of the bladder or urinary tract should be ruled out, either by direct visualization with a cystoscopy, or by imaging. The concomitant onset of bladder symptoms can suggest the involvement of the urinary tract. Symptoms may include pneumaturia, recurrent urinary tract infections, and increased urinary frequency.

Preoperatively, an enema may be performed to empty the lower colon, however full bowel preparation is not necessary. We utilize the enhanced recovery protocol for all patients undergoing abdominal surgery [25]. The preferred surgical approach to abdominal fistula repair is a minimally invasive one, using robotic-assisted laparoscopy. If there is underlying colon pathology, such as diverticulitis or prior pelvic radiation, a bowel resection with primary anastomosis may be necessary. In cases of radiation, a diverting stoma should also be performed due to a higher risk of anastomotic leak. In this scenario, the sigmoid colon is mobilized along the retrorectal space distal to the fistula site. Once the fistulous tract is identified, the distal end of the colon is mobilized and stapled circumferentially. A colpotomy is performed and the diseased colonic segment removed, either through the colpotomy, or through an extended umbilical incision. An end-to-end primary anastomosis is then performed with a circular stapler. The colpotomy is closed using a delayed absorbable suture. An omental flap can be mobilized and interposed between the rectal anastomosis and the vagina.

**Figure 5:**
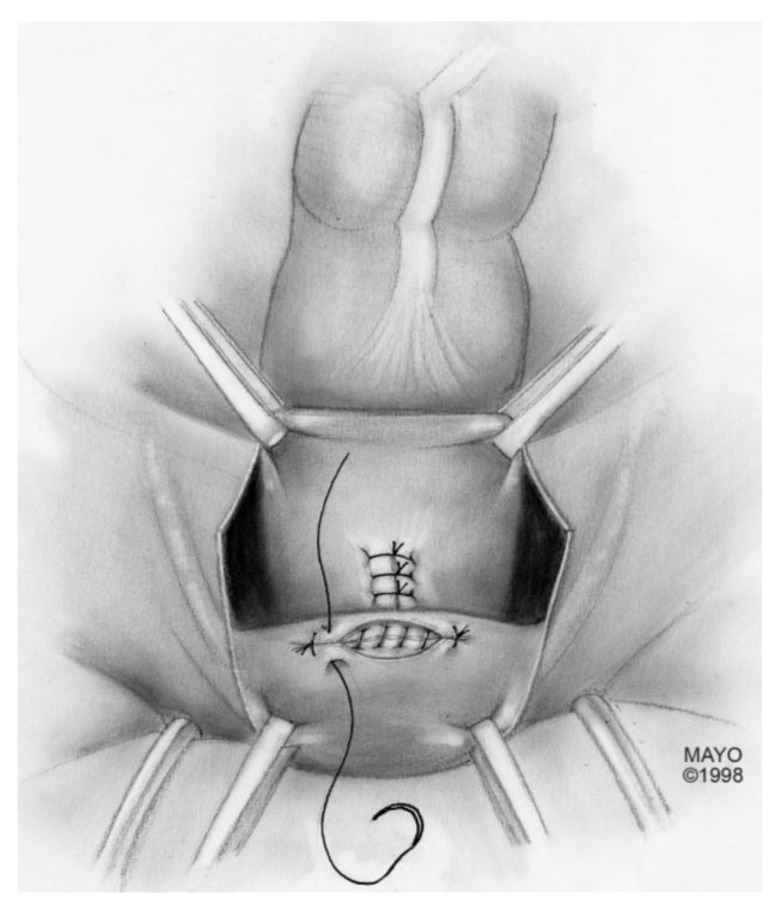
Anatomical illustration demonstrating technique of surgical repair of high rectovaginal fistulas.

If the fistula is iatrogenic due to gynecologic surgery, bowel resection may not be necessary. In this scenario, the rectovaginal space is carefully opened until the fistulous tract is identified (Figure 5). The tract is excised until healthy vaginal and rectal tissue is identified. A discoid excision of the rectum is thus performed and primarily closed, using a delayed absorbable suture, in a multilayer fashion. The colpotomy is also closed with a delayed absorbable suture. An omental flap can be interposed in the rectovaginal space with the omentum sutured distal to the colpotomy and rectotomy sites.

The appropriate anatomical identification of the fistula is crucial in order to determine the extent of the surgery, and to be able to counsel the patient as to the best surgical approach to be taken.

## 3. Percutaneous Novel Interventional Techniques

Percutaneous interventional techniques are continuously evolving, and provide a new armamentarium for patients with fistulas who are deemed not to be surgical candidates. Although surgical options exist, including resection with primary anastomosis, resection with diversion, or diversion alone, a select group of patients may not be operative candidates due to other medical comorbidities. Endoscopic endoluminal stenting has been considered as an alternative option, as covered stents can effectively palliate the symptoms by secluding the vagina from the colon [26]. For complex surgical patients, new technologies are being designed so that percutaneous interventional methods can be used to offer hope and an improved quality of life for these patients.

**Figure 6:**
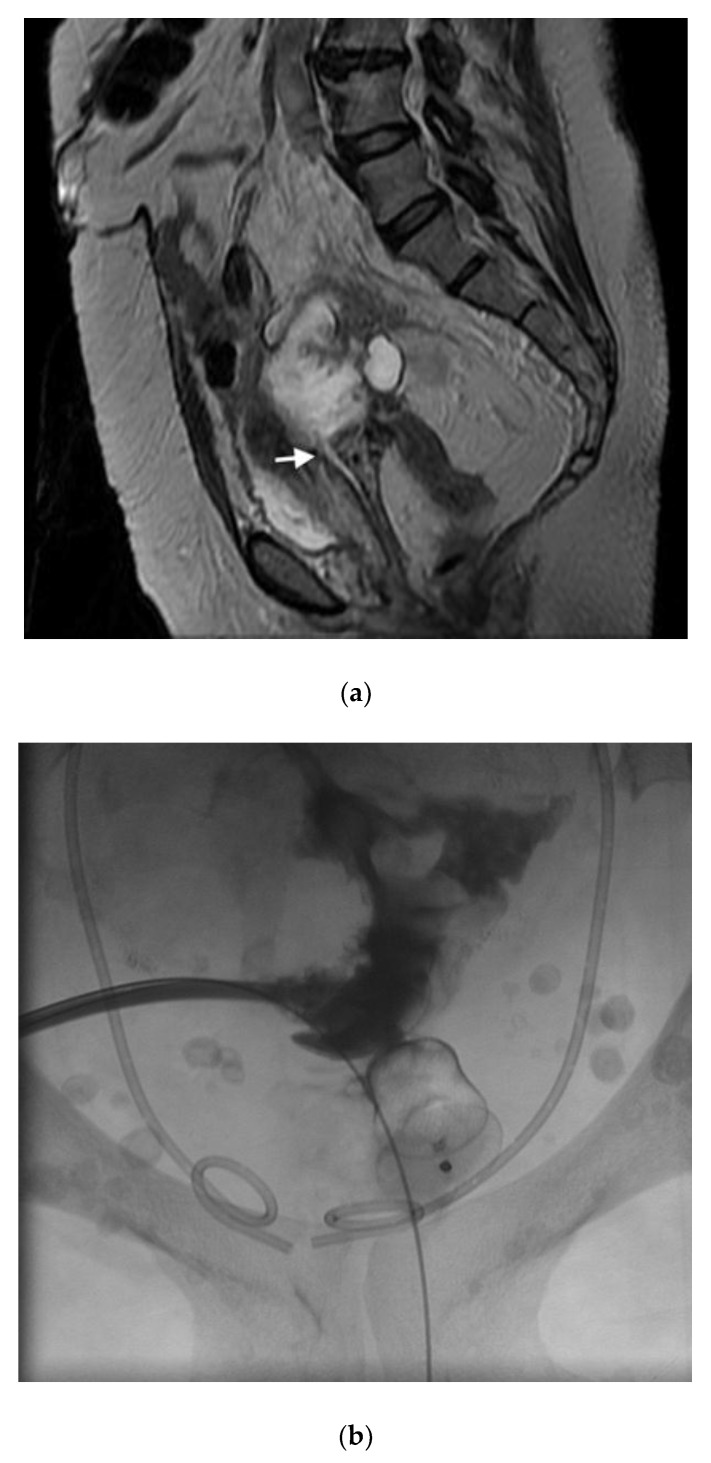
(**a**) Sagittal T2 MR imaging shows communication between a pseudomyxomatous tumor and the vagina (arrow); (**b**) contrast injection shows no further communication between the tumor and the vagina just after deployment of the Amplatzer 2 plug.

In the literature pertaining to interventional radiology, enterocutaneous fistulas have been closed using the Biodesign Fistula Plug (Cook, Inc., Bloomington, IN, USA), however this plug has limited sizes, and may not be adequate for closing large vaginal fistulas. Thus far in the literature, there are only a handful of reports which have used the AMPLATZER occluder device (AVP; St. Jude Medical, Inc. St. Paul, MN, USA) for closure of rectovaginal fistulas (Figure 6) [27,28]. These reports offer future promise for adapting current interventional devices and/or designing new technologies. In the first case report, Kilickesmez used the AVP for treating a rectovaginal fistula, which occurred following a low anterior resection for rectal cancer. Prior surgical interventions, namely diversion colostomy and primary surgical repair were both unsuccessful. An AVP 2 (AVP; St.Jude Medical, Inc. St. Paul, MN, USA) was used to occlude the fistula, and was deployed percutaneously through the vaginal lumen. Although initially successful in this case, long-term success was not achieved, likely attributable to contractile forces within this area [27]. Lee et al. designed their own fistula plug for the occlusion of rectovaginal fistulas, consisting of a cloverleaf-configurated disk with a nitinol frame [20]. They placed this device transvaginally in seven patients with either radiation induced or surgically induced fistulas, achieving good outcomes, even up to 26 months following the procedure. In a more recent article, the AMPLATZER plug was used to create an occlusive barrier across a carcinoid mucinous tumor-vaginal fistula, with good short-term outcomes.

**Figure 7:**
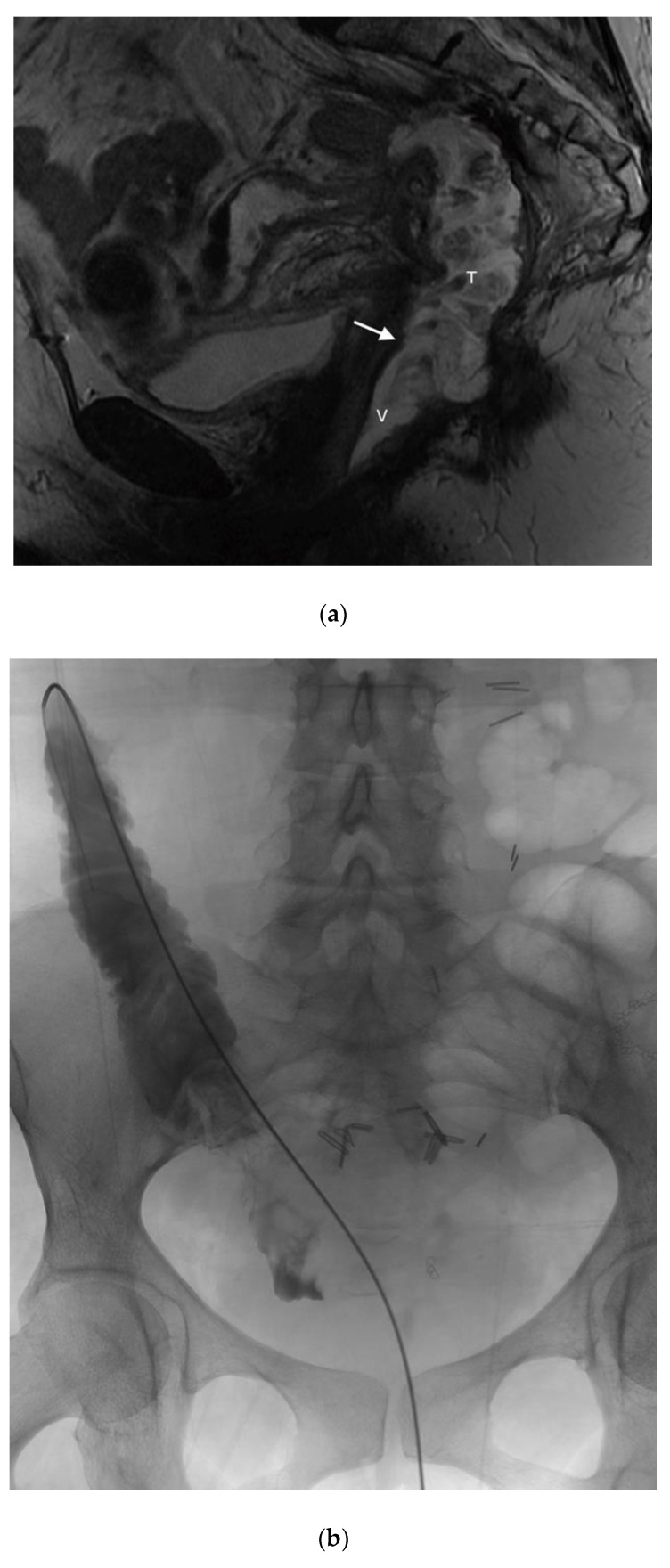

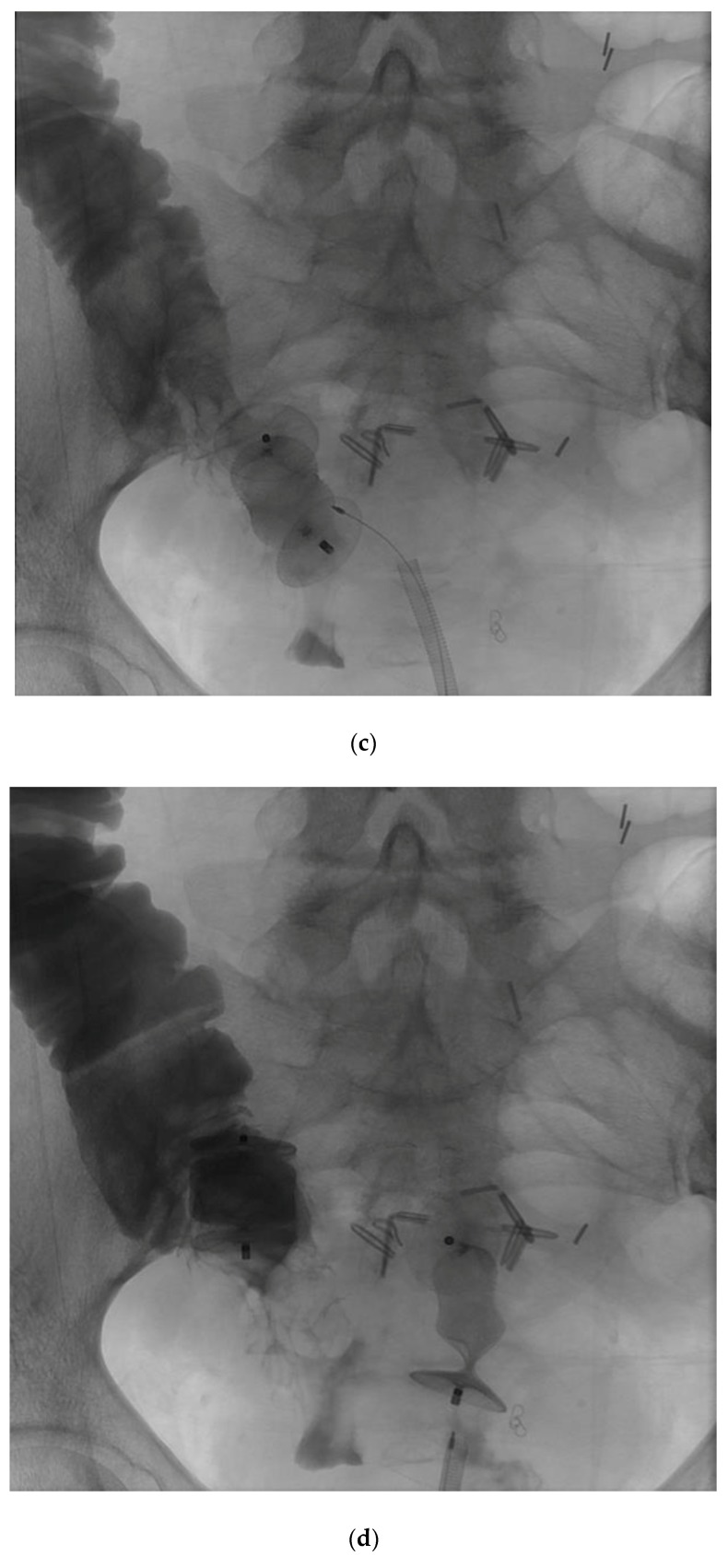

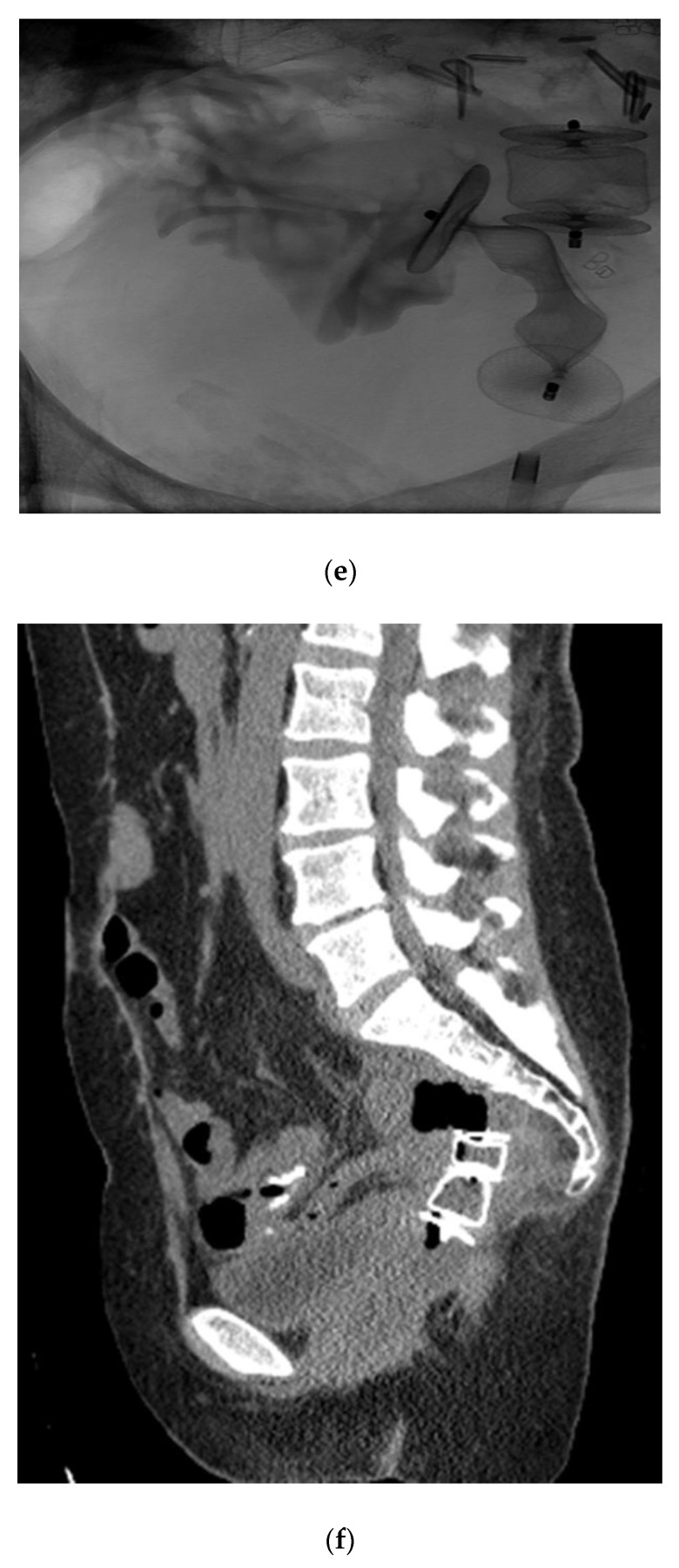
(**a**) Sagittal T2 MR shows a large fistula (arrow) between a recurrent mucinous rectal tumor (T) and the vagina (V). The rectum is surgically absent; (**b**) a guidewire has been passed through the vagina into the fistula with the tumor and colon. Contrast is seen opacifying the cecum and ascending colon; (**c**) the first Amplatzer device is being deployed; (**d**) the second Amplatzer device is being deployed; (**e**) after the Amplatzer device is deployed, contrast is retained in the small bowel and no longer communicates with the vagina; (**f**) a sagittal CT image shows the two adjacent Amplatzer plugs within the fistula tract.

Given the successful short-term outcome, a second patient was referred for the closure of a complex colovaginal fistula resulting from invasive vaginal malignancy and prior surgical intervention. The patient was deemed not to be a candidate for operative repair. The patient had such a significantly poor quality of life that percutaneous closure of the fistula was attempted (Figure 7).

The patient did well in the short term, having a significant decrease in vaginal output, and an improved quality of life. Furthermore, follow-up imaging demonstrated that the occluder plugs were in the appropriate position.

## 4. Conclusions

Vaginal fistulas are rare complications resulting from various disease processes or surgical interventions. However, they can and should be treated in order to dissipate distressing symptoms in women. The majority of these women can be treated through surgical intervention, using the treatment approaches described above. However, for those women who are not surgical candidates, and who experience a significantly reduced quality of life as a result of these fistulas, endovascular interventions can be applied. Although a promising treatment option, long-term follow-up is needed for these patients in order to ensure the long-term occlusion of the fistulas.
